# Enzymology and Regulation of δ^1^-Pyrroline-5-Carboxylate Synthetase 2 From Rice

**DOI:** 10.3389/fpls.2021.672702

**Published:** 2021-09-14

**Authors:** Giuseppe Sabbioni, Dietmar Funck, Giuseppe Forlani

**Affiliations:** ^1^Department of Life Science and Biotechnology, University of Ferrara, Ferrara, Italy; ^2^Plant Physiology and Biochemistry Laboratory, Department of Biology, University of Konstanz, Konstanz, Germany

**Keywords:** proline biosynthesis, substrate affinity, product inhibition, enzyme regulation, redox status, NADPH/NADP^+^ ratio

## Abstract

Under several stress conditions, such as excess salt and drought, many plants accumulate proline inside the cell, which is believed to help counteracting the adverse effects of low water potential. This increase mainly relies upon transcriptional induction of δ^1^-pyrroline-5-carboxylate synthetase (P5CS), the enzyme that catalyzes the first two steps in proline biosynthesis from glutamate. P5CS mediates both the phosphorylation of glutamate and the reduction of γ-glutamylphosphate to glutamate-5-semialdehyde, which spontaneously cyclizes to δ^1^-pyrroline-5-carboxylate (P5C). In most higher plants, two isoforms of P5CS have been found, one constitutively expressed to satisfy proline demand for protein synthesis, the other stress-induced. Despite the number of papers to investigate the regulation of P5CS at the transcriptional level, to date, the properties of the enzyme have been only poorly studied. As a consequence, the descriptions of post-translational regulatory mechanisms have largely been limited to feedback-inhibition by proline. Here, we report cloning and heterologous expression of P5CS2 from *Oryza sativa*. The protein has been fully characterized from a functional point of view, using an assay method that allows following the physiological reaction of the enzyme. Kinetic analyses show that the activity is subjected to a wide array of regulatory mechanisms, ranging from product inhibition to feedback inhibition by proline and other amino acids. These findings confirm long-hypothesized influences of both, the redox status of the cell and nitrogen availability, on proline biosynthesis.

## Introduction

Water stress tolerance is a major goal for plant genetic improvement, and most likely a need to secure crop productivity threatened by ongoing climate changes (Ahanger et al., 2017). As soon as a lowering in soil water potential hinders water absorption at the root level, plants react by closing stomata and activate a series of mechanisms for osmotic compensation, among which a pivotal role is played by the accumulation of the so-called compatible osmolytes (Sharma et al., 2019). These osmoprotectants include sugars (Iordachescu and Imai, 2008), tertiary amines (Annunziata et al., 2019), and amino acids (Hildebrandt, 2018). Among amino acids, accumulation of the cyclic amino acid proline is the most widespread in plants and other organisms (Verbruggen and Hermans, 2008; Meena et al., 2019). However, there are a number of reports indicating that the concentrations of accumulated proline are not high enough to contribute significantly to osmotic adjustment (e.g., Forlani et al., 2019a). Free proline is rapidly accumulated not only in response to drought and salinity, but also to cold, heavy metals, or pathogen attack (Hayat et al., 2012). Because of this variety of inducing stimuli, several other beneficial effects of proline have been hypothesized, ranging from stabilization of membranes and enzymes to reactive oxygen species scavenging, regulation of redox balance, or induction of antioxidant defenses (Liang et al., 2013; Kishor and Sreenivasulu, 2014; Govrin et al., 2019; Forlani et al., 2019b).

Whatever the exact, multifaceted role in the plant response to adverse conditions, stress-induced accumulation of proline has been shown to depend mainly upon an increase of its biosynthetic rate (Trovato et al., 2019). Several pathways have been described for the biosynthesis of this amino acid, being those from glutamate or ornithine as the major routes (Fichman et al., 2015). In bacteria, proline synthesis from glutamate is accomplished by three enzymes: γ-glutamyl kinase (EC 2.7.2.11) that catalyzes glutamate phosphorylation, NADPH-dependent γ-glutamyl phosphate reductase (EC1.2.1.41) that reduces the product to glutamate semialdehyde, which in solution spontaneously cyclizes to δ^1^-pyrroline-5-carboxylate (P5C), and P5C reductase (EC1.5.1.2) that produces proline, using either NADH or NADPH as the electron donor (Chen et al., 2006; Csonka and Leisinger, 2007; Forlani et al., 2017). In higher plants, the first two steps were found to be catalyzed by a single bifunctional enzyme bearing both catalytic domains of glutamyl kinase and glutamyl-phosphate reductase, P5C synthetase (P5CS; Hu et al., 1992; Rai and Penna, 2013). Another plant enzyme able to synthesize glutamate semialdehyde was similarly isolated by trans-complementation of *proBA* mutants (Delauney et al., 1993). Because of the occurrence of such an ornithine-δ-aminotransferase (EC 2.6.1.13) and of the finding that mammalian P5CS is feedback-inhibited by ornithine (Hu et al., 1999), it was hypothesized that under conditions of osmotic stress and nitrogen limitation proline synthesis in plants proceeds through the glutamate pathway, while the ornithine pathway assumes prominence under high nitrogen input (Delauney and Verma, 1993; Anwar et al., 2018). Although some confirmatory results have been reported (Da Rocha et al., 2012), other findings have shown that ornithine-derived P5C is oxidized to glutamate in the mitochondrion (Funck et al., 2008; Winter et al., 2015), and – at least in *Arabidopsis* – proline biosynthesis is now believed to proceed exclusively *via* the glutamate pathway. Consistently, induction of *P5CS* was found in virtually all cases in which proline accumulation occurred in response to the exposure to stress conditions (Trovato et al., 2019).

Isolation and sequencing of *P5CS* led to the identification of two isogenes in several plant species and phylogenetic analyses demonstrated that *P5CS* duplication occurred independently in several taxonomic groups (Turchetto-Zolet et al., 2008; Rai and Penna, 2013, Fichman et al., 2015). These paralogs (named P5CS1 and P5CS2) showed non-overlapping roles, with varying temporal and spatial expression patterns. In *Arabidopsis thaliana*, *AtP5CS1* is expressed in most plant organs, whereas *AtP5CS2* is predominantly expressed in areas of active cell division (Strizhov et al., 1997; Székely et al., 2008). The analysis of GFP-fused proteins suggested partial chloroplastic localization of PCS1, whereas P5CS2:GFP was predominantly found in the cytosol (Székely et al., 2008). However, more recent data conclusively demonstrated a cytosolic localization of both isoenzymes (Funck et al., 2020). Transcription of the *P5CS* genes was differentially induced by drought, salinity, and abscisic acid and, consistently, the occurrence of a wide but different array of putative transcription factor binding sites was shown in the promoters of the two paralogs (Fichman et al., 2015; Zarattini and Forlani, 2017).

Knockout *p5cs1* mutants showed reduced proline synthesis under stress, hypersensitivity to salt stress, and accumulation of reactive oxygen species, whereas *p5cs2* mutations caused embryo abortion during late stages of seed development (Székely et al., 2008). Germination and establishment under ambient conditions, but not susceptibility to infection with *Pseudomonas syringae*, were impaired in *p5cs2* mutants, whereas stress-induced proline accumulation was much less affected than in *p5cs1* mutants (Funck et al., 2020). Overall, AtP5CS1 has therefore been identified as the major contributor to stress-induced proline accumulation, whereas AtP5CS2 plays a pivotal role in embryo development and growth. The rice (*Oryza sativa*) genome also contains two *P5CS* isogenes, which seem to have a somehow less defined role. *P5CS1*, located on chromosome 1, was induced by stress conditions, such as the exposure to salt, dehydration, and cold. *P5CS2*, mapping on chrosomome 5, was expressed in mature plant parts, yet it was also induced by salt and mannitol treatments (Rai and Penna, 2013; Forlani et al., 2015a).

Contrary to the large amount of information available on the regulation of *P5CS* gene expression in different plant tissues, during development and in response to environmental signals, very little is known about the biochemical features of P5CS isoenzymes and the occurrence of post-translational regulatory mechanisms. The activity of a single enzyme cloned from *Vigna aconitifolia* showed 50%-inhibition at 6mM proline (Hu et al., 1992). The activity of the same enzyme and of a proline-insensitive form obtained by site-directed mutagenesis was characterized in more detail afterward, showing *K*_m_ values for glutamate and ATP of 3.6 and 2.7mM, respectively (Zhang et al., 1995). However, these data were obtained using an assay method that measures only the partial glutamyl kinase activity, and not the full forward reaction catalyzed by the enzyme. Another study on the effect of osmotic stress on P5CS activity in *Arabidopsis* leaves used quantification of the inorganic phosphate released after glutamyl-phosphate reduction (Parre et al., 2010). An assay that follows the glutamate- and ATP-dependent oxidation of NADPH was used to analyze the activity of a recombinantly expressed putative P5CS from tomato, which has later been identified as a bacterial contamination in the cDNA library used (García-Ríos et al., 1997). A similar assay has also been used to demonstrate the activity of P5CS overexpressed in switchgrass (*Panicum virgatum*; Guan et al., 2019). However, proper controls were not performed in any of these studies to test if the detected activity was influenced by the presence in the crude extracts of glutamine synthetase (EC 6.3.1.2), which also catalyzes the phosphorylation of glutamate and releases phosphate in the presence of even low concentrations of ammonia (Forlani et al., 2006). To the best of our knowledge, neither kinetic analysis of plant P5CS activity has been reported, nor the functional features of P5CS isozyme pairs have been investigated and compared so far. As a consequence, some long lasting hypotheses linking modulation of proline biosynthesis with either the nitrogen status (Delauney et al., 1993; Delauney and Verma, 1993) or the redox status (Sharma et al., 2011; Shinde et al., 2016) of the cell still await substantiation. Here, we report cloning, recombinant expression, purification, and functional characterization of P5CS2 from rice, providing a first body of evidence toward this goal.

## Materials and Methods

### Cloning, Heterologous Expression, and Purification of Rice P5CS2

The coding sequence of *O. sativa* P5C synthetase 2 (locus Os05g38150; protein ID BAG95649) was amplified by PCR from cDNA clone J033099M14 (RGRC-NIAS) with the primers P5CS2-fw (caccATGGCGAGCGTCGACCCGT) and P5CS2-rev (agcatttgaccTCATTGCAAAG), and inserted by directional TOPO cloning into the expression vector pET151 that provides an N-terminal His_6_-tag (Life Technologies, Carlsbad, CA, United States). For heterologous expression, *Escherichia coli* BL21(DE3) pLysS cells (Invitrogen, Carlsbad, CA, United States), made competent by the calcium chloride method, were transformed with the resulting vector pET151-*Os*P5CS2. Transformants were selected on LB plates containing 100mgL^−1^ ampicillin and 50mgL^−1^ chloramphenicol. After inducing the expression of the plant protein at 24°C by the addition of 1mM IPTG to liquid LB medium, the cells were harvested by centrifugation and extracted by either sonication or grinding with 2gg^−1^ alumina in a mortar at 4°C in extraction buffer (50mM Na phosphate buffer, pH 7.5, containing 200mM NaCl, 5mM DTT, and 20mM imidazole). The clarified extract was loaded at a constant flow of 10mlh^−1^ onto a His-SpinTrap™ Nickel Sepharose Gel column (GE Healthcare, Little Chalfont, United Kingdom; 0.1ml bed volume) for purification of the His-tagged protein. Stepwise washing and elution was achieved by increasing concentrations of imidazole in extraction buffer, while collecting 1-ml fractions. For activity assays, the purified enzyme was diluted into assay buffer (see below).

Alternatively, cells were extracted in 20mM Tris-HCl buffer, pH 7.5, containing 5mM DTT. Following centrifugation for 5min at 10.000*g*, the soluble extract was desalted by passage through a BioGel P6DG (BioRad) column, and loaded onto a DEAE-Sephacel (Pharmacia) column (1.5cm diameter, 10ml bed volume) equilibrated with the same buffer at a constant flow of 30mlh^−1^. The column was then eluted with a linear gradient from 0 to 250mM NaCl (200ml), for the collection of 4-ml fractions.

The His_6_-tag was cleaved by treatment with His-tagged TEV protease (final concentration 0.1mgml^−1^) for 20min at pH 7.5. To remove TEV protease and the cleaved fragment, a negative chromatography on the same His-SpinTrap™ Nickel Sepharose Gel column was carried out after diluting the sample to lower imidazole concentration. Protein concentration was determined by the method of Bradford (1976), using bovine serum albumin as the standard. Enzyme preparations were stored at 4°C in the dark.

### P5C Synthetase Assay

#### Glutamyl Kinase Assay

Glutamyl kinase assay was performed as described previously (Campanile et al., 1993), with minor modifications. Enzyme preparations (about 2μg protein) were incubated up to 30min at 30°C with 20mM glutamate, 4mM ATP, and 100mM hydroxylamine hydrochloride in 50mM Tris-HCl buffer, pH 7.5, in a final volume of 75μl. The reaction was terminated by the addition of 150μl of a colorimetric mixture consisting of 10% (w/v) FeNO_3_×9 H_2_O, 6.67% (v/v) HCl, and 5% (w/v) trichloroacetic acid. Following centrifugation for 3min at 12,000*g*, samples were read at 535nm against non-incubated blanks. The γ-glutamyl-hydroxamate formed was quantified on the basis of a calibration curve obtained with an authentic standard.

#### Glutamyl Phosphate Reductase Assay

To determine glutamyl phosphate reductase activity (Campanile et al., 1993), enzyme preparations (about 2μg protein) were incubated up to 20min at 30°C with 2mM DL-P5C, 5mM NADP^+^, and 10mM K_2_HPO_4_ in 50mM Tris-HCl buffer, pH 7.5, in a final volume of 200μl. The P5C-dependent formation of NADPH was followed by reading the samples at 1min intervals at 340nm against blanks in which P5C had been omitted. The amount of NADPH formed was quantified on the basis of a calibration curve obtained with an authentic standard. DL-P5C was synthesized by the periodate oxidation of δ-*allo*-hydroxylysine and purified by cation exchange chromatography on a 200–400 mesh Dowex AG50W-X4 column, as previously described (Forlani and Funck, 2020).

#### NADPH Oxidation Assay

The physiological, forward reaction of P5C synthetase was measured by following the glutamate-dependent oxidation of NADPH in the presence of ATP. Aliquots of the purified enzyme preparations (0.5–1μg protein) were incubated in a final volume of 200μl with 20mML-glutamate, 4mM ATP, and 0.4mM NADPH in 50mM Tris-HCl buffer, pH 7.5. When aiming at the evaluation of the effect of chlorides on enzyme activity, Tris-HCl buffer concentration was lowered to 10mM, or replaced with 10mM potassium phosphate buffer, pH 7.5. Incubation proceeded at 30°C for up to 20min. The decrease of absorbance at 340nm was determined at 0.5min intervals against exact blanks in which glutamate had been omitted. Assays were either performed in cuvettes with 0.2 or 1cm pathlength (UVette; Eppendorf, Milan, Italy), or in 96-microwell plates. In the former case, OD_340_ was determined with a Novaspec plus spectrophotometer (Amersham Biosciences, Milan, Italy) equipped with an UVette adaptor. In the latter case, the plate was equilibrated at 30°C prior to enzyme addition, and absorbance was measured using a Ledetect plate reader (Labexim, Lengau, Austria) equipped with a LED plugin at 340nm. Each sample was carried out in triplicate (technical replications). Each determination was repeated with at least three different enzyme preparations (biological replications). Presented data refer to a single enzyme preparation, and are means±SE over technical replicates. Linear and non-linear regressions of data, as well as kinetic constant values, were computed using Prism 6 for Windows, version 6.03 (GraphPad Software, San Diego, CA, United States).

### Analytical Methods

For SDS-PAGE analysis, samples were denatured by boiling for 5min in a treatment buffer consisting of 2% (w/v) SDS, 10% (v/v) glycerol, and 5% (v/v) β-mercaptoethanol in 62.5mM Tris–HCl buffer, pH 6.8. Sample aliquots (10–40μl) were subjected to discontinuous sodium dodecyl sulfate (SDS)-polyacrylamide gel electrophoresis (PAGE) at 8mA with a 5% stacking and a 10% separating gel. For total protein analysis, *E. coli* cells were harvested by centrifugation and directly resuspended in treatment buffer at 10 optical units (600nm) ml^−1^. For soluble protein analysis, *E. coli* cells were harvested by centrifugation, resuspended in 20mM Tris-HCl buffer (pH 7.5) at 20 optical units (600nm) ml^−1^, and sonicated with five 1-min pulses at 70% amplitude with a UP50H ultrasonic processor (Hielsher Ultrasonic, Teltow, Germany). Samples were centrifuged again, and the supernatant was treated with the same volume of 2X treatment buffer. Gels were stained for proteins with Quick Coomassie Stain (Neo Biotech, Nanterre, France).

Inclusion bodies were isolated by differential centrifugation according to Upadhyay et al. (2012). Solubilization of proteins from inclusion bodies was performed with the methods reported by Singh et al. (2015).

To obtain peptide mapping, the purified protein was subjected to SDS-PAGE and then excised, destained, digested with both trypsin and pepsin, and analyzed by reversed phase liquid chromatography-tandem mass spectrometry using an Esquire 3000 spectrometer (Bruker Daltonics), connected to an Agilent 1100 HPLC. After sample injection, the column was washed for 5min with 90% mobile phase A (0.1% formic acid) and 10% mobile phase B (0.1% formic acid in acetonitrile), and peptides were eluted using a linear gradient from 10 to 80% mobile phase B in 20min at 50μlmin^−1^. The Esquire mass spectrometer was operated in a data-dependent mode in which each full MS scan was followed by three MS/MS scans where the three most abundant molecular ions were dynamically selected and fragmented by collision-induced dissociation. Dynamic exclusion was allowed.

## Results

### Heterologous Expression of *Oryza sativa* P5C Synthetase 2 in *E. coli* and Affinity Purification of the Catalytically Active Protein

**Figure 1:**
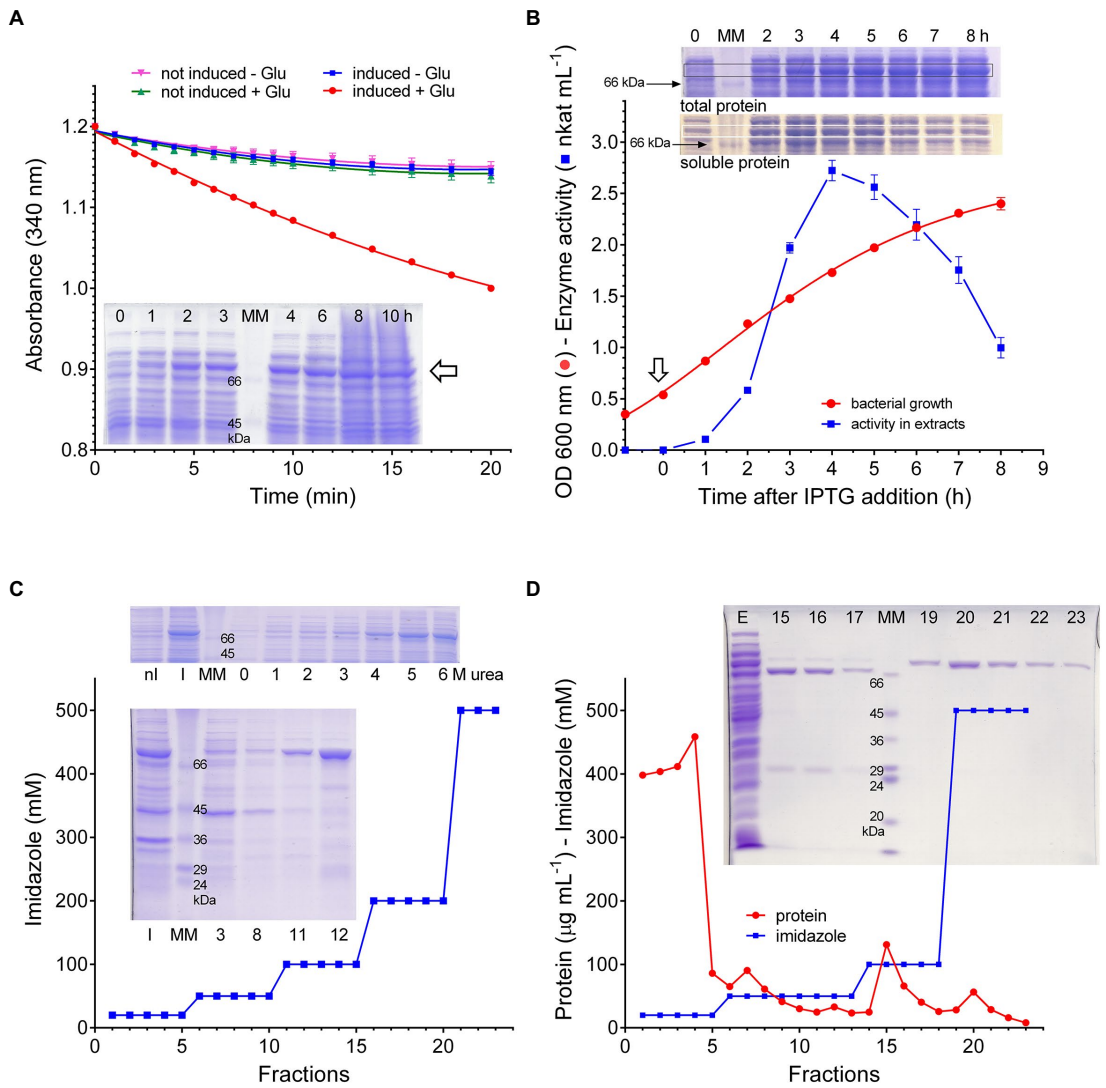
Expression of rice *P5CS2* in *Escherichia coli*. Following the treatment with IPTG, extracts were prepared from bacterial cells transformed with the vector pET151-*Os*P5CS2. The measurement of NADPH oxidation in extracts prepared 4h after induction showed the presence of a significant rate that was not evident neither in the absence of glutamate nor in extracts from non-induced cells. SDS-PAGE analysis of total protein from the same cultures, harvested at increasing time as indicated, disclosed the appearance of a new band (⇦) with a relative molecular mass (80kDa) that was compatible with that expected for the recombinant protein **(A)**. When the time-course of the glutamate-dependent NADPH oxidation was measured, the attainment of maximal specific activity values was found 4–5h after induction (⇩); thereafter, activity levels decreased progressively with time. Data are mean±SE over triplicates. SDS-PAGE analysis of parallel samples, shown as insets, revealed a similar pattern for the 80kDa band among soluble proteins; on the contrary, once the maximal levels was reached, it remains subsequently constant among total proteins **(B)**. Inclusion bodies were isolated by differential centrifugation from extracts from induced *E. coli* cells, and treated with increasing concentration of urea. SDS-PAGE analysis of the supernatant of the samples in this way obtained showed quantitative solubilization of the 80kDa band at urea levels exceeding 4M. Affinity chromatography in the presence of 6M urea allowed the recovery of substantial levels of this protein, which eluted from the Ni^++^-agarose column at 100mM imidazole. The number of the lanes in the lower gel refers to the fraction of the eluate that was analyzed; nI, inclusion bodies from non-induced cells; I, induced cells; and MM, molecular weight markers **(C)**. Cell-free extracts were prepared from bacterial cells harvested 4h after IPTG treatment, and extracts were loaded onto a Ni^++^-agarose column. Proteins were then eluted by a stepwise imidazole gradient, and fractions were analyzed by SDS-PAGE. The samples eluted with 500mM imidazole contained a single, homogeneous band with a relative molecular mass of about 80kDa. Also in this case, numbers of the lanes in the gel refer to the fractions of the eluate that were analyzed; E, extract; MM, molecular weight markers **(D)**. All experiments were repeated three times on independent samples (biological replications), and very similar results were obtained.

The gene coding in rice for the stress-induced P5CS isoenzyme 2 (protein ID O04226) was cloned into the expression vector pET151 and used to transform *E. coli* BL21(DE3) pLysS cells. Following the treatment with IPTG, glutamate and ATP-dependent NADPH oxidation activity was detectable in crude extracts, which was not present in extracts from either non-induced cells or induced cells transformed with the empty vector. Simultaneously, a pronounced band of the predicted molecular mass (81.5kDa) became evident upon SDS-PAGE analysis of total protein samples prepared at increasing time after IPTG addition (Figure 1A). Preliminary attempts to purify the plant protein from induced cells harvested 10–24h after induction failed, since negligible protein amounts were found in the eluate from Nickel Affinity Gel columns. To understand the reason for such a failure, the presence of the protein was investigated by gel electrophoresis of samples obtained by either directly resuspending bacterial cells with denaturing treatment buffer (total protein), or using supernatants obtained after cell sonication (soluble protein). Results showed that in total protein samples the band of interest reached a maximal amount about 4h after induction, and remained constant thereafter. On the contrary, after reaching maximal amounts in a similar timeframe, its presence among soluble proteins declined at increasing time to lowest levels. Consistent patterns were obtained when specific activity levels of glutamate and ATP-dependent NADPH oxidation were measured in cell free extracts from parallel samples (Figure 1B). Because results were suggestive of a fast and complete sequestering of the heterologous protein in inclusion bodies, induction was performed at lower temperatures, down to 15°C, but this did not lead to substantial improvements. As an alternative approach, protein solubilization from inclusion bodies was performed. Several treatments were tried with this aim, such as 5% (v/v) DMSO or *n*-propanol or 0.5–2% (w/v) n-dodecyl-β-D-maltoside, but the best results were obtained with urea: extraction with 5–6M urea allowed a quantitative recovery of the protein in the supernatant, and the inclusion of 6M urea into column buffers afforded satisfactory yields from the subsequent affinity chromatography step (Figure 1C). However, despite the many attempts by means of dilution, dialysis, or column desalting protocols, renaturation of purified samples did not provide an active protein. Active P5CS2 protein was obtained only when non-denaturing extracts were prepared from cells harvested 3–5h after IPTG treatment, when bacterial cultures were still in the mid-exponential phase of growth. Even in this case, the attainment of homogeneous preparations required a careful setup of elution conditions from the affinity column, and extensive washing with buffers containing 50 and 100mM imidazole was necessary to resolve the plant enzyme from contaminant *E. coli* proteins that were also retained by the resin (Figure 1D).

The identity of the purified protein was confirmed by peptide mapping, from which a 55%-coverage was obtained (Supplementary Figure S1). The His_6_-tail was removed by treatment with TEV protease and subsequent negative chromatography on the Nickel Affinity Gel column to get rid of the detached fragment and the protease. No differences were found in the enzymological features of the protein before and after the cleavage. Therefore, the removal was not carried out subsequently, also to avoid a consequent, partial loss of specific activity. The purified enzyme was in fact extremely unstable, with a half-time of less than 24h at 4°C (data not shown). Every attempt to identify stabilizing conditions failed. The only treatment that slightly increased the stability of recombinant P5CS2 was the addition of high concentrations of a reducing agent (dithiothreitol 5–10mM) to extraction and column buffers, although in the latter case levels higher than 5mM could not be used in order to avoid interference with the affinity gel. In the absence of dithiothreitol, activity was completely lost within 24h. To overcome such drawback, small amounts of the protein were purified each day, and used for the characterization of the functional properties of the enzyme within 4–5h after the isolation.

### Activity Assays and Substrate Affinities

**Figure 2:**
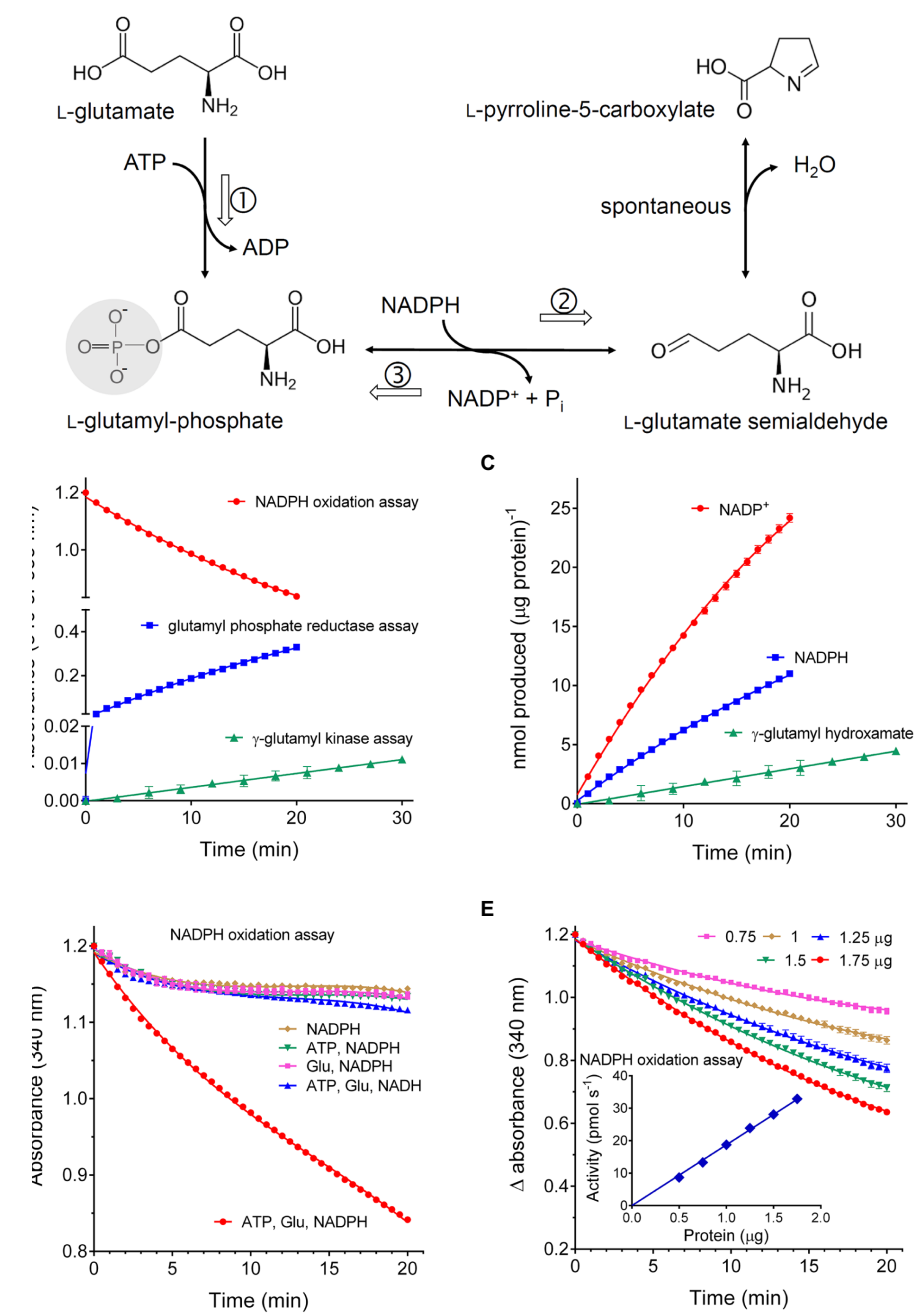
Activity of recombinant rice P5C synthetase 2. **(A)** shows the reaction catalyzed by the enzyme: following glutamate phosphorylation and ADP release, glutamyl-phosphate is reduced using NADPH as the electron donor, yielding glutamate semialdehyde that spontaneously cyclizes to P5C. Three assay methods have been described: the glutamate kinase assay, which measures the formation of γ-glutamyl-hydroxamate in the absence of NADPH (①); the glutamyl-phosphate reductase assay, which follows the reverse, P5C- and Pi-dependent reduction of NADP^+^ in the absence of glutamate (③); the NADPH oxidation assay in the presence of all three substrate, thereby following the full forward, physiological reaction of the enzyme (①+②). The purified protein was assayed with these three methods using aliquots corresponding to 2, 2, and 1μg protein, respectively **(B)**. To obtain a quantitative comparison of the three methods, data in **(A)** were used to calculate the absolute amounts of the product formed in each case *per* μg of protein **(C)**. The NADPH oxidation assay was performed by incubating 1μg of the purified protein with 0.4mM NADPH in the presence of an array of all possible combinations of the other substrates. A mixture containing ATP and NADH instead of NADPH was also included. Non-limiting levels of all three substrates were required to maintain the rate of NADPH oxidation over time **(D)**. Because an initial burst of NADPH consumption was evident also in the absence of ATP and glutamate, the activity of increasing amounts of protein was measured against exact blanks in which glutamate had been omitted **(E)**. The inset shows that in this case activity values, calculated by interpolation of the linear rate of NADPH oxidation, were strictly proportional to the amount of the enzyme. In all cases, results are means±SE over three technical replications. Each experiment was repeated three times with different enzyme preparations, obtaining very similar patterns.

Rice P5CS2 was at first assayed with two colorimetric methods that had been previously described in the literature, i.e., the phosphorylation of glutamate in the presence of product-trapping hydroxylamine (glutamyl kinase assay), and the reverse, P5C-dependent reduction of NADP^+^ in the presence of inorganic phosphate (glutamyl phosphate reductase assay). In both cases, activity was detectable when 1–2μg of purified protein was incubated at 30°C up to 30min. However, due to the low molar extinction coefficient of the glutamyl-hydroxamate assay (760 A_535_ M^−1^ cm^−1^), very low absorbance values were obtained in the former case. In the latter, though the change in absorbance was much greater (*ε*=6,220 A_340_ M^−1^ cm^−1^), the activity rapidly lost linearity and was not proportional to the amount of enzyme, most likely because of the reversibility of the reaction. On the contrary, when all the three substrates (glutamate, ATP, and NADPH) were incubated with the purified protein (NADPH oxidation assay), a nearly linear rate of NADPH oxidation was detected at 340nm (Figure 2A), which was significantly higher than both rates of the partial activities when data were expressed on a molar basis (Figure 2B). The simultaneous production of P5C was also verified (Supplementary Figure S2). Because this method measures in addition the full, physiological reaction, it was used thereafter throughout the characterization of the enzymological features of the enzyme. The presence of non-limiting ATP and glutamate concentrations was required to maintain a linear reaction rate over time. However, an initial burst of NADPH oxidation was evident even in the absence of one or both of the other substrates (Figure 2C). If equimolar amounts of NADPH were oxidized, similar results could imply the occurrence of a biphasic mechanism in which a rapid reaction of NADPH with the enzyme is followed by a slower steady-state process, in which glutamyl phosphate is produced and subsequently reduced to P5C. On the contrary, the amount of NADPH oxidized largely exceeded that of the enzyme in the reaction mixture, and the transfer of electrons to an enzyme-bound cofactor was not supported by the UV-vis spectrum of the purified protein (Supplementary Figure S3). The possibility therefore exists that such an initial burst may depend on a trace compound introduced with the enzyme or the buffer, which is rapidly reduced by NADPH. In any case, following a pre-incubation with the dinucleotide before the addition of glutamate and ATP, the resulting rate of NADPH oxidation was linear, and the rates in the stationary state with or without pre-incubation were comparable (Supplementary Figure S4). No detectable utilization of NADH was found, even at the highest concentration tested (0.5mM). For the subsequent estimation of the steady state rate and the assessment of biochemical parameters and functional features, each sample was therefore read at 30s intervals for 20min against an exact blank in which glutamate had been omitted. The resulting difference in absorbance decreased almost linearly with time, and was strictly proportional to the amount of enzyme (Figure 2D). Under such standard assay conditions, a mean specific activity of 15.92±0.43 nkat mg^−1^ protein (*n*=20) was found.

**Figure 3:**
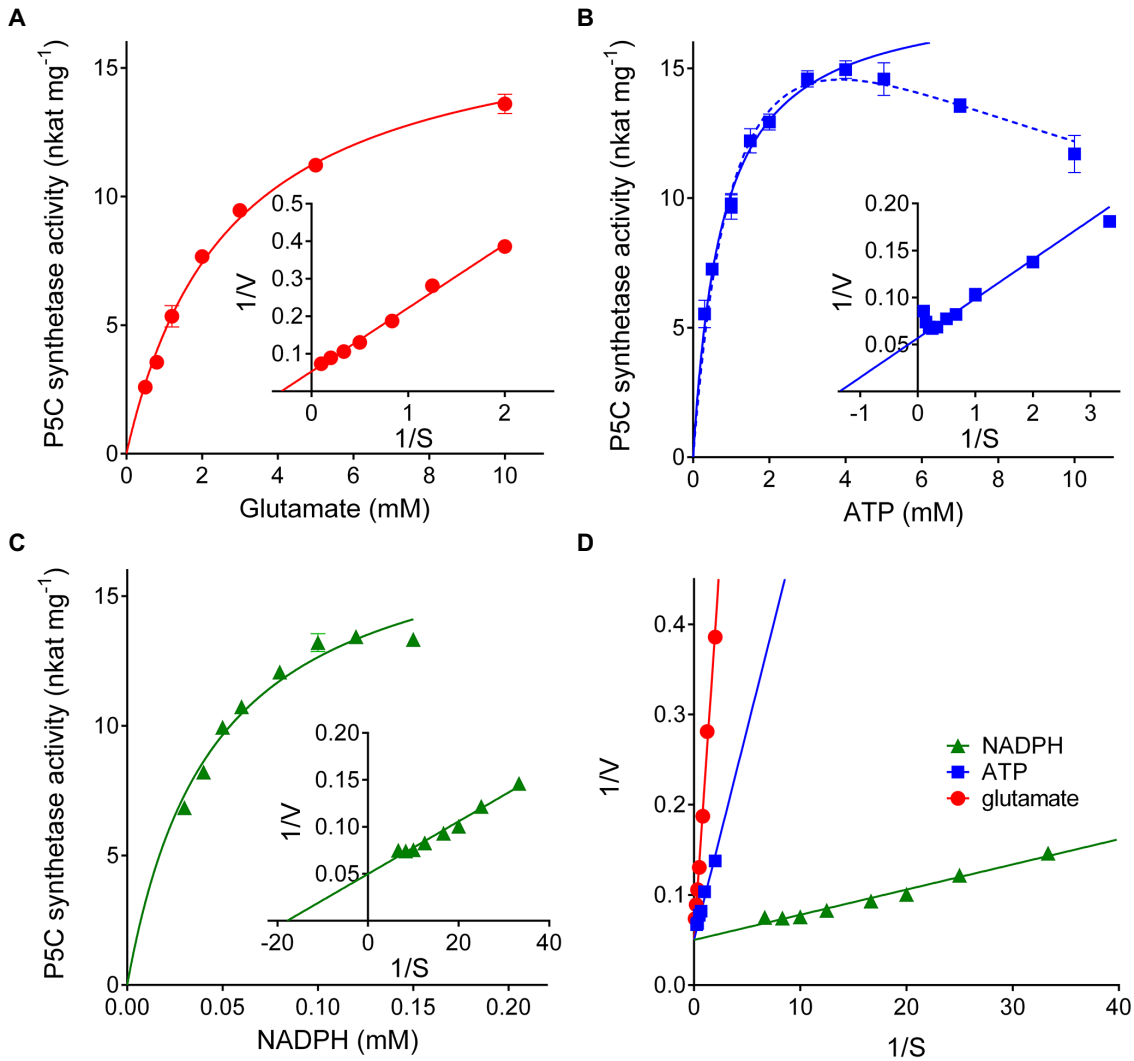
Substrate affinity and catalytic properties of rice P5C synthetase 2. Enzyme activity was measured by the NADPH oxidation assay at varying the concentration of glutamate **(A)**, ATP **(B)**, or NADPH **(C)** while maintaining the fixed concentrations of the other substrates (20mM glutamate, 4mM ATP, and 0.4mM NADPH). Lineweaver-Burk plots of data (the inset in each panel) allowed calculation of apparent affinities and *V*_max_ values (Table 1). Presented results are means±SE over three technical replicates. The experiments were repeated three times with different enzyme preparations, obtaining very similar values. In panel **(B)**, the solid line refers to non-linear interpolation obtained by considering ATP concentrations up to 4mM, whereas the dashed line is that obtained by considering all the experimental values using a substrate inhibition model. To gain information on the reaction mechanism of this multisubstrate enzyme, a combined Lineweaver-Burk graph was plotted **(D)**.

Substrate affinities were evaluated by varying the concentration of a single substrate while maintaining the other substrates at saturating concentrations (20mM glutamate, 4mM ATP, and 0.4mM NADPH, Figures 3A–C). Lineweaver-Burk plots of the results obtained in this way allowed estimation of apparent *K*_M_ values (Table 1). Consistent results were calculated in all cases for the maximal catalytic rate, with a *V*_max_ value of about 18nkat (mg protein)^−1^, which corresponds to only 1.5 catalytic events s^−1^. Significant enzyme activity required the presence of 1–10mM glutamate, 0.1–1mM ATP, and 10–100μM NADPH, but ATP concentrations exceeding 4mM were found inhibitory (Figure 3B). Fitting the data to a substrate inhibition model (*Y*=*V*_max_*X/(*K*_M(app)_+X*[1+*X*/*K*_I_]), where *V*_max_ is the maximum enzyme velocity, if the substrate did not also inhibit enzyme activity, and *K*_I_ is the dissociation constant for substrate binding in such a way that two substrates can bind to an enzyme; equation 5.44 in Copeland, 2000) allowed calculation of a *K*_I_ value for ATP of 11.6±5.1mM. Multisubstrate enzymatic reactions may proceed through two different mechanisms, a sequential process in which the binding of all substrates must take place before the release of products, and a non-sequential or “ping-pong” mechanism in which one substrate binds, and one product is released before a second substrate binds, and a second product is released. Converging lines in a global Lineweaver-Burk plot (Figure 3D) are considered supportive of the former possibility. However, additional experiments to study the variation of apparent *V*_max_ and *K*_M_ while varying other substrates are needed to confirm the nature of the mechanism.

**Table 1:** Kinetic properties of P5C synthetase 2 from rice.

Vmax (Glu)	17.38±0.52	nkat (mg protein)^−1^
Vmax (ATP)	17.96±0.46	nkat (mg protein)^−1^
Vmax (NADPH) steady-state rate	18.36±0.64	nkat (mg protein)^−1^
KM(app) for L-Glu	2.69±0.19	mM
KM(app) for ATP^*^	0.764±0.067	mM
KM(app) for NADPH steady-state rate	0.045±0.004	mM

* *Value calculated from the Lineweaver-Burk plot of results obtained by considering ATP concentrations≤4mM. When all the results shown in*Figure 3B*were considered using the inhibition model, a value of 1.29±0.20 was instead found*.

### Product and Feed-Back Inhibition by Proline, Proline Analogues, and Metabolically Related Amino Acids

**Figure 4:**
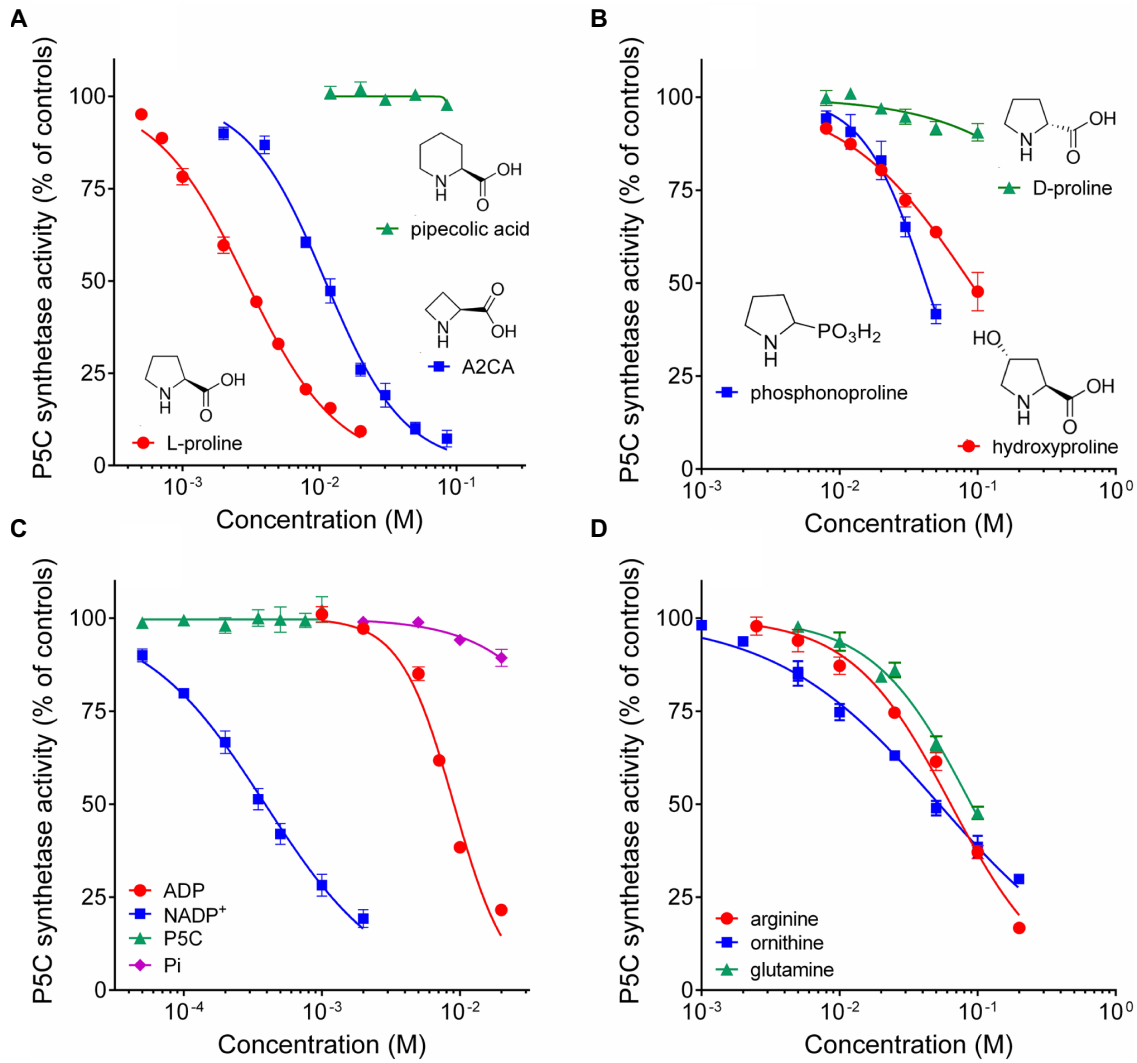
Effect of reaction products and related amino acids on the activity of rice P5C synthetase 2. The activity of the purified protein was assayed in the presence of increasing concentrations of the final product of the pathway, L-proline, and its 4C (A2CA) and 6C (pipecolic acid) analogues **(A)**; of the D-isomer and other natural and synthetic analogues of proline **(B)**; of the four enzyme products **(C)**; of some amino acids that are metabolically interconnected with proline, or represent a cellular index of nitrogen availability **(D)**. Results were expressed as per cent of mean values in untreated controls. Presented data are means±SE over three technical replicates. Each experiment was repeated three times with different enzyme preparations, and virtually identical patterns were obtained. Ornithine and arginine were added as hydrochlorides. To rule out the possibility that effects may depend on a consequent change in pH and not to the added substances, the actual pH in each sample was measured with a microelectrode at the end of the incubation. Non-linear regression of data (log[inhibitor] *vs*. normalized response – variable slope) was performed using GraphPad Prism; IC_50_ values and their confidence intervals are reported in Table 2.

To evaluate the possible occurrence of post-translational regulative mechanisms, rice P5CS2 was assayed in the presence of increasing concentrations of products, analogues, and intermediates in proline synthesis, as well as other amino acids that are metabolically related, or represent an index of the nitrogen status of the cell. As expected, millimolar levels of proline were found inhibitory (Figure 4A), with a concentration inhibiting activity by 50% (IC_50_) of less than 3mM (Table 2). The four-atom ring analogue of proline, azetidine-2-carboxylate (A2CA), similarly inhibited P5CS2 activity, although with a 3.8-fold higher IC_50_. Pipecolate, which differs from proline by an additional C-atom in the ring, was found completely ineffective in the range tested (Figure 4A). Up to 100mM, D-proline was unable to interfere with the activity of P5CS2, whereas hydroxyproline and phosphonoproline showed partial inhibition (Figure 4B). On the whole, these results suggest that similarly-sized structural analogues of L-proline are able to bind to P5CS2 and mimic the feed-back inhibition of the enzyme.

**Table 2:** Concentrations of selected substances able to inhibit by 50% the activity of *Oryza sativa* P5C synthetase 2.

	IC_50_ (mM)	Lower limit (mM)	Upper limit (mM)
L-proline	2.91	2.76	3.08
azetidine-2-carboxylic acid	11.0	10.2	11.9
phosphonoproline	42.2	37.8	47.3
L-4-hydroxyproline	89.5	76.4	105
NADP^+^	0.389	0.365	0.414
ADP	9.06	8.40	9.77
ornithine	52.6	48.4	57.2
arginine	63.9	58.0	70.3
glutamine	89.7	83.7	96.2
Mg^++^	34.8	27.6	46.4
Ca^++^	4.09	3.60	4.63

*Confidence intervals (p=0.05) are also reported*.

Concerning the possibility that the P5CS2 may be subjected to product inhibition, the addition of micro to millimolar levels of P5C or inorganic phosphate to the assay mixture did not exert any significant effect. On the contrary, a remarkable inhibition was found in the case of both NADP^+^ and ADP (Figure 4C). The latter was inhibitory only at concentrations exceeding that of ATP in the reaction mixture (4mM), and 9mM ADP was required to reduce activity by 50%. More interestingly, NADP^+^ exerted a significant inhibition already at levels 5-fold lower than those of NADPH in the assay, and 50% inhibition was achieved when NADP^+^ and NADPH were present at the same concentration (0.4mM). NAD^+^ was completely ineffective (not shown). The results therefore strengthen the possibility that P5CS activity may be regulated *in vivo* by both the adenylate charge and, mainly, by the redox status of the cell.

The effect of the presence of other amino acids was also analyzed. The addition of concentrations of ornithine exceeding 2mM was found inhibitory (Figure 4D), and 50%-inhibition of P5CS activity was obtained at about 50mM (Table 2). A similar pattern was evident also with arginine, though the effect was slightly lower. Enzyme activity was affected also by glutamine at concentrations exceeding 10mM, with an IC_50_ value of about 100mM. Asparagine and α-ketoglutarate were on the contrary ineffective up to 100mM (data not presented).

### Effect of Anions and Cations on Enzyme Activity

**Figure 5:**
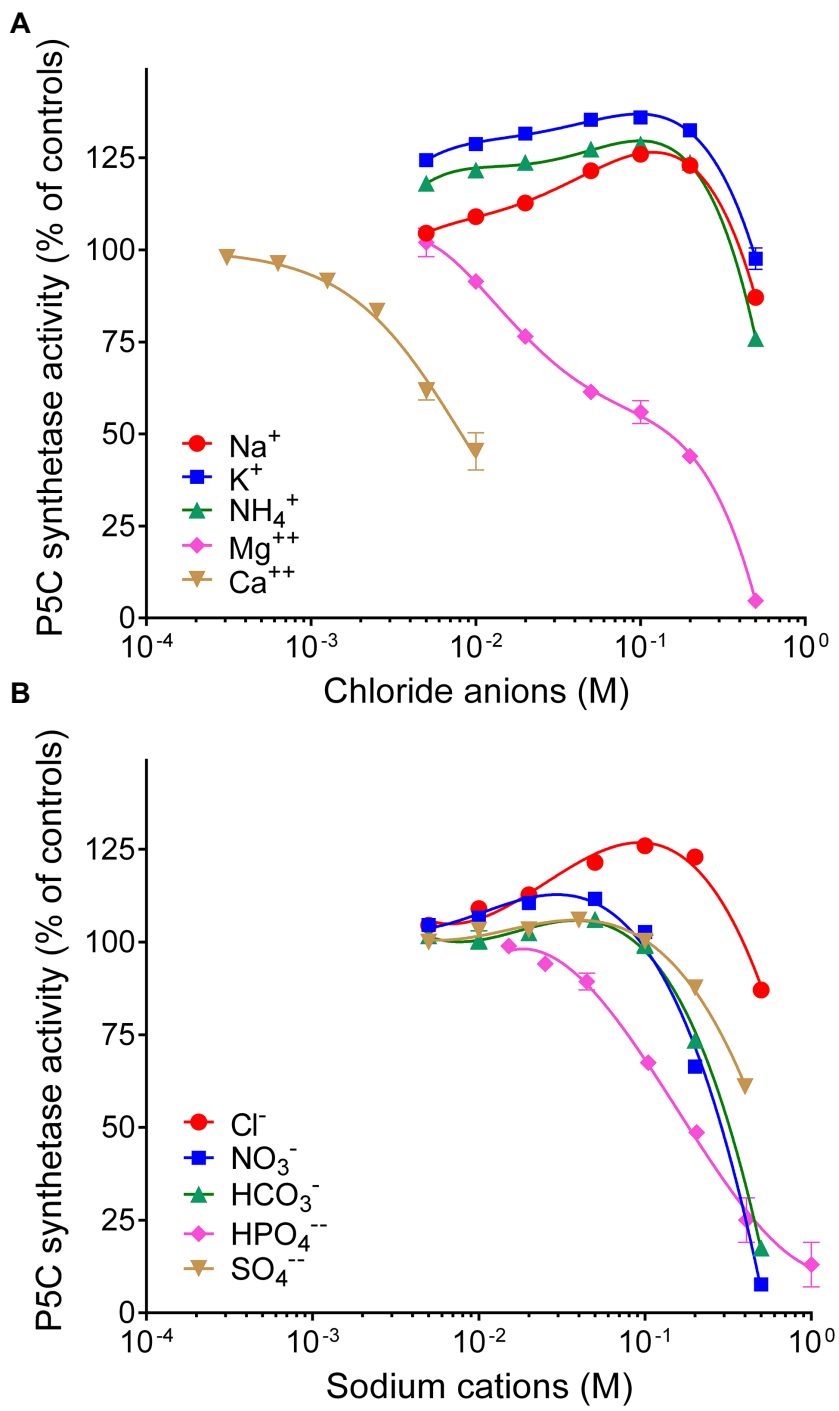
Effect of anions and cations on the activity of rice P5C synthetase 2. The steady state rate of the enzyme was measured in the presence or in the absence of increasing concentrations of chlorides of various cations **(A)** or the sodium salts of various anions **(B)**, ranging from 0.3mM to 1M. To minimize ion presence in the assay mixture, in the former case, Tris-HCl buffer was replaced with 10mM potassium phosphate buffer, pH 7.5, whereas in the latter case, its concentration was lowered to 10mM. The actual pH in each sample was measured with a microelectrode at the end of the incubation, to ensure that the addition of salts did not cause a pH change in the reaction mixture. Activity was expressed as per cent of mean value in untreated controls. Data are means±SE over three technical replicates. The experiments were repeated three times with different enzyme preparations, and almost identical patterns were obtained. To calculate the concentration of Mg^++^ ions able to inhibit activity by 50%, data were expressed with respect to parallel samples containing equimolar amounts of NaCl, to compensate the stimulatory effect of chlorides in the range 10–200mM.

Finally, because the synthesis of proline is often induced by the exposure to hyperosmotic conditions, the effect of the main anions and cations present in plant cells on the activity of P5CS was investigated (Figure 5). When considered overall, data suggested a stimulatory effect of chloride anions in the range 10–100mM. On the contrary, sodium cations seem inhibitory, but only at concentrations exceeding 100mM. Among the other anions, HPO_4_^−−^ reduced P5CS activity when added to the reaction mixture at levels higher than 20mM. Unexpectedly, a strong inhibitory effect was evident with divalent Ca^++^ and Mg^++^. Ca^++^ was more effective, although it had only a negligible effect at concentrations typically present in the cytosol. Mg^++^ ions at 20mM, a level that had been routinely added to the γ-glutamyl kinase assay (Zhang et al., 1995), exerted 25% inhibition (Figure 4A), and 50%-inhibition was found at about 35mM (Table 2).

## Discussion

Recombinant expression of rice P5CS isoenzyme 2 in *E. coli* and subsequent affinity purification to electrophoretic homogeneity yielded an active protein, but the activity was highly unstable. In the absence of reducing agents, the half-life of P5CS2 activity was less than 24h. This feature is in striking contrast to the remarkable stability of the enzyme that catalyzes the second and last step in proline biosynthesis, P5C reductase, which retained more than 90% activity after 3-month storage at 4°C (Giberti et al., 2014; Ruszkowski et al., 2015; Forlani et al., 2015b). Another remarkable difference between the two enzymes concerns their catalytic constants, with P5CS2 catalyzing only 1.5 reactions s^−1^ per monomer, whereas rice P5C reductase showed the capability of reducing 350 to almost 5,000 P5C molecules s^−1^ under non-limiting conditions, depending on whether NADPH or NADH was the electron donor (Forlani et al., 2015b). Such a huge difference may be functional *in vivo* to avoid any accumulation of P5C, which has been hypothesized to exert (or trigger) cytotoxic effects (Deuschle et al., 2004; Qamar et al., 2015; Giberti et al., 2017), and could explain the lack of correlation between the expression level of P5C reductase and the intracellular concentration of proline (Hua et al., 1997). Moreover, a high lability of the enzyme that catalyzes the limiting step in a biosynthetic route may facilitate a rapid activation or deactivation of the pathway as a function of the cellular need for the final product. However, more evidence is required to confirm that P5CS2 may have a similar lability also inside the plant cell, where microenvironmental conditions or posttranslational modifications could improve its half-life. Both *A. thaliana* P5CS isoenzymes were found to be phosphorylated *in vivo* (PhosPhAt database; Durek et al., 2010) but no information is available about the influence of these modifications on the stability or activity of P5CS.

Proline biosynthesis from glutamate is competitively feedback-inhibited by proline binding near the glutamate binding site of the γ-glutamylkinase domain of P5CS, and Hill coefficients of 1.7–2 indicated cooperativity between proline binding to individual γ-glutamylkinase active sites (Zhang et al., 1995; Pérez-Arellano et al., 2010; Fichman et al., 2015). The activity of purified rice P5CS2 was inhibited by 50% at about 3mM proline, and less than 20% catalytic rate was retained at 10mM proline. These values are similar to the reported proline inhibition of the γ-glutamyl kinase activity of P5CS from *V. aconitifolia*, and roughly 20 times higher than the IC_50_ of *E. coli* γ-glutamyl kinase (Zhang et al., 1995; Pérez-Arellano et al., 2010). Moderate sensitivity to proline inhibition seems consistent with the role hypothesized for class 2 P5CS isozymes, i.e., to provide the steady-state intracellular levels of proline required for protein synthesis (Rai and Penna, 2013). A phenylalanine residue (F133 in OsP5CS2) that was found critical for feedback inhibition of plant P5CS (Zhang et al., 1995) is conserved in both P5CS isoenzymes in rice, as well as in *Arabidopsis*. It remains to be analyzed, if differences at other positions render the stress-inducible isoforms less sensitive to inhibition by proline in order to allow proline accumulation. Interestingly, the 4C-ring analogue A2CA was also found to interact with rice P5CS2, thereby exerting a *false* feedback that was early hypothesized to explain part of the phytotoxic effects of this naturally-occurring compound (Nielsen et al., 1986).

The activity of rice P5CS was measured with three methods that had been described in the literature: by following the ATP-dependent phosphorylation of glutamate in the absence of NADPH (glutamyl kinase assay), the P5C-dependent reverse reduction of NADP^+^ (glutamyl phosphate reductase assay), and the complete forward reaction in the presence of all three substrates (NADPH oxidation assay). The rate of glutamyl phosphate production was much lower than that of the whole reaction. This could depend on the lability of γ-glutamyl phosphate (Hayzer and Leisinger, 1980) even in the presence of product-trapping hydroxylamine. The alternative possibility that a slow kinase reaction is favored by a faster release of ADP in the presence of NADPH would imply a “ping-pong” mechanism for this multisubstrate enzyme. On the contrary, the occurrence of an initial burst of NADPH oxidation even in the absence of glutamate and ATP strengthens a biphasic mechanism in which a rapid reaction of NADPH with the enzyme is followed by a slower process in which glutamyl phosphate is produced and reduced. Considering that the glutamyl phosphate reductase assay is not sensitive to feedback inhibition by proline (Zhang et al., 1995; Supplementary Figure S4), these data emphasize the need of adopting the NADPH oxidation assay to obtain a reliable estimate of the biochemical features of P5CSs. However, the presence of several unrelated activities able to hydrolyze NADPH, coupled with the low specific activity of P5CS2, makes this assay poorly feasible if used with crude extracts. Accordingly, knockout of one P5CS isoform had only a small effect on total ATP- and glutamate-dependent NADPH oxidation in total protein extracts of *Medicago truncatula* (Nguyen et al., 2013). With the NADPH oxidation assay, the purified rice P5CS2 showed a *K*_m_ value for glutamate similar to those described previously, whereas the *K*_m_ value calculated for ATP (0.76mM) was significantly lower than those reported for the half-reactions (glutamyl kinase assay) of the enzymes from *V. aconitifolia* (2.7mM; Zhang et al., 1995) or *A. thaliana* (1.5mM; Parre et al., 2010). Substrate inhibition was also found at ATP concentrations exceeding 4mM. If we convert literature data (25–35nmolg^−1^ fw; e.g., Yu et al., 2020) into molar levels on the assumption of an intracellular volume (excluding vacuoles) equal to about 2–4% of the total water content in rice leaves, the concentration of ATP in the cell should range from 0.6 to 1.5mM. It is therefore likely that such inhibition does not have a physiological role. Our kinetic analysis of purified rice P5CS2 also enabled determination of the apparent *K*_m_ for NADPH as 45μM, which had not been determined before for any plant P5CS.

The actual concentration of NADPH in the plant cell is not easy to be estimated, as literature data are expressed in nmol (g FW)^−1^ (Queval and Noctor, 2007; Takahashi et al., 2009; Sharma et al., 2011). However, these values should correspond to cytosolic concentrations of NADPH ranging from 50 to 100μM. This would imply that every fluctuation of the NADPH level could influence the rate of P5C synthesis. Taking into account that the exposure to stress conditions is well-known to induce cytosolic NADPH production through the oxidative pentose phosphate pathway (OPPP; Baxter et al., 2007), this would also explain the significant increase of proline homeostatic levels that rapidly occurred in rice cell cultures after the exposure to hyperosmotic stress even in the absence of any variation in P5CS expression levels (Forlani et al., 2015a). Indeed, following a treatment with PEG at −1.2MPa, NADPH levels in 7-day-old seedlings of *A. thaliana* increased from 0.3 to 2nmol (g FW)^−1^ (i.e., from about 15 to 100μM; Sharma et al., 2011). A higher rate of proline synthesis would in turn regenerate NADP^+^, allowing sustained OPPP activity (Hare and Cress, 1997; Verslues and Sharma, 2010). Interestingly, we found that the activity of rice P5CS2 is also sensitive to product inhibition, with 50% reduction of the catalytic rate in the presence of equimolar levels (0.4mM) of NADP^+^ and NADPH. An even higher sensitivity to NADP^+^ has been found for plant P5C reductases, but only if NADH was the electron donor, whereas the NADPH-dependent activity was unaffected (Giberti et al., 2014; Ruszkowski et al., 2015; Forlani et al., 2015b). The almost complete inability of rice P5CS2 to use NADH as cofactor indicates a strong interaction with the phosphate group of NADPH, which may explain why also NADP^+^ may be bound strongly and thus inhibit NADPH binding. Structures of human P5CS (2H5G) and glutamyl phosphate reductase from yeast (1VLU) are available, but they do not include NADPH. NADP^+^ levels in *Arabidopsis* usually range from 2 to 25nmol (g FW)^−1^, approximatively corresponding to 0.1–1.25mM; Queval and Noctor, 2007; Takahashi et al., 2009; Sharma et al., 2011). If similar values were present also in rice, this would imply that under nonstressful conditions P5CS2 activity is significantly inhibited by NADP^+^, but promptly activated under stress as a consequence of the above-mentioned reduction of the pyridine dinucleotide pool. Mechanisms linking the modulation of proline biosynthesis with the redox status of the cell have long been hypothesized (Hare and Cress, 1997; Sharma et al., 2011; Shinde et al., 2016). Characterization of rice P5CS2 provides for the first time useful elements to substantiate this hypothesis.

Also noteworthy is the finding that rice P5CS2 is inhibited not only by proline, but also by ornithine, arginine, and glutamine. In mammals, P5CS is not feedback-inhibited by proline, but its activity is regulated by ornithine, with a *K*_I_ value (0.4mM) very near the predicted intracellular concentration of the intermediate (Hu et al., 1999). However, in mammals, the P5CS reaction serves both proline and arginine biosynthesis, whereas in plants, the first committed step of arginine production is the acetylation of glutamate, yielding a product that is then subjected to phosphorylation and reduction steps analogous to those catalyzed by P5CS (Winter et al., 2015). This notwithstanding, it has been hypothesized that the glutamate pathway is the main route for proline synthesis in plants only under nitrogen limitation, whereas under high nitrogen input the ornithine pathway assumes prominence (Delauney and Verma, 1993; Anwar et al., 2018). Although the occurrence of the ornithine pathway has been questioned in *Arabidopsis* (Funck et al., 2008), the sensitivity of P5CS not only to ornithine, but also to amino acids that are abundant under high nitrogen input might provide the molecular basis of such regulative mechanism. P5CS2 was inhibited *in vitro* by ornithine and arginine concentrations higher than those found in rice cultured cells [4–15nmol (g FW)^−1^ and 15–76nmol (g FW)^−1^ for ornithine and arginine, which should correspond to about 0.1–0.4 and 0.4–4mM, respectively; Forlani et al., 2014]. However, higher concentrations of ornithine, arginine, and glutamine are found in certain plant tissues as nitrogen storage forms [e.g., 0.4, 5, and 35μmol (g FW)^−1^, respectively, in 12day-old *Arabidopsis* seedlings Majumdar et al., 2016; or 14, 53, and 9μmol (g DW)^−1^, respectively, in shoots of fertilized plants of *Vaccinium vitis-idaea* Ann-Brittedfast et al., 1994], and inside the cell cumulative effects also are likely to occur. Therefore, inhibition of P5CS activity by other amino acids than proline may have a physiological role under certain conditions.

On the other hand, the addition to the reaction mixture of chlorides in the 10–100mM range was found to apparently stimulate P5CS2 activity up to 40%. This effect might be functional to proline accumulation under hyperosmotic stress. A similar effect has been shown to a much higher extent (up to 4-fold) also for P5C reductase, but only when NADPH was the electron donor (Forlani et al., 2015b). In *E. coli*, glutamate binding to the active site of γ-glutamylkinase is stabilized by an inter-subunit hydrogen bond network and it is perceivable that such a network may be sensitive to subtle changes in the quaternary structure brought about by altered ion concentrations in the cytosol (Pérez-Arellano et al., 2010). Crystallographic or mutational studies will be required to determine the effects of ion binding to P5CS. Contrary to *A. thaliana*, where mostly the P5CS1 isoform mediates stress-induced proline accumulation, both P5CS isoforms are important for proline accumulation and stress tolerance in rice (Hien et al., 2003; Hur et al., 2004; Forlani et al., 2015a). Indeed, to obtain a comprehensive picture of the post-translational regulation of the glutamate pathway for proline synthesis, the elucidation of functional properties of a P5CS family-1 member is also required. Work is currently in progress in our laboratory to fill this gap through cloning, heterologous expression, purification, and characterization of rice P5CS1.

## Data Availability Statement

The raw data supporting the conclusions of this article will be made available by the authors, without undue reservation.

## Author Contributions

GF designed the study, performed part of the experiments, elaborated the results, and drafted the paper. GS purified the P5CS2 protein and performed part of the experiments. DF cloned the *P5CS2* gene and performed peptide mapping. All authors contributed to the article and approved the submitted version.

## Funding

This work was supported by grants from the University of Ferrara (FAR 2019 and 2020).

## Conflict of Interest

The authors declare that the research was conducted in the absence of any commercial or financial relationships that could be construed as a potential conflict of interest.

## Publisher’s Note

All claims expressed in this article are solely those of the authors and do not necessarily represent those of their affiliated organizations, or those of the publisher, the editors and the reviewers. Any product that may be evaluated in this article, or claim that may be made by its manufacturer, is not guaranteed or endorsed by the publisher.
