# Supplementary material for: Enzymology and Regulation of δ1-Pyrroline-5-Carboxylate Synthetase 2 From Rice
Source: Front Plant Sci. 2021 Sep 14;12:672702. doi: 10.3389/fpls.2021.672702 (PMC8480329; doi:10.3389/fpls.2021.672702)
Supplement: Supplementary file 1 [file Data_Sheet_1.PDF]

1 11 21 31 ↓ 41 51 61
MHHHHHXGKP IPNPLLGLDS TENLYFQGID PFTMASVDPS RSFVRDVKRV IIKVGTAVVS RQDGRLALGR

71 81 91 101 111 121 131
VGALCEQVKE LNSLGYEVIL VTSGAVGVGR QRLRYRKLVN SSFADLQKPQ MELDGKACAA VGQSGLMALY

141 151 161 171 181 191 201
DMLFNQLDVS SSQLLVTDSD FENPKFREQL TETVESLLDL KVIPIFNEND AISTRKAPYE DSSGIFWDND

211 221 231 241 251 261 271
SLAGLLALEL KADLLILLSD VDGLYSGPPS EPSSKIIHTY IKEKHQQEIT FGDKSRVGRG GMTAKVKAAV

281 291 301 311 321 331 341
LASNSGTPVV ITSGFENRSI LKVLHGEKIG TLFHKNANLW ESSKDVSTRE MAVAARDCSR HLQNLSSEER

351 361 371 381 391 401 411
KKILLDVADA LEANEDLIRS ENEADVAAAQ VAGYEKPLVA RLTIKPGKIA SLAKSIRTLA NMEDPINQIL

421 431 441 451 461 471 481
KKTEVADDLV LEKTSCPLGV LLIVFESRPD ALVQIASLAI RSGNGLLLKG GKEAIRSNTI LHKVITDAIP

491 501 511 521 531 541 551
RNVGEKLIGL VTTRDEIADL LKLDDVIDLV IPRGSNKLVS QIKASTKIPV LGHADGICHV YIDKSADMDM

561 571 581 591 601 611 621
AKHIVMDAKI DYPAACNAME TLLVHKDLMK SPGLDDILVA LKTEGVNIYG GPIAHKALGF PKAVSFHHEY

631 641 651 661 671 681 691
SSMACTVEFV DDVQSAIDHI HRYGSAHTDC IVTTDDKVAE TFLRRVDSAA VFHNASTRFS DGARFGLGAE

701 711 721 731 741
VGISTGRIHA RGPVGVEGLL TTRWILRGRG QVVNGDKDVV YTHKSLPLQ

FIGURE S1 | Peptide mapping of *Oryza sativa* P5C synthetase 2. Following SDS-PAGE, the purified protein was destained, excised, digested with both trypsin and pepsin and analyzed by reversed phase liquid chromatography-tandem mass spectrometry. The Esquire mass spectrometer was operated in a data-dependent mode in which each full MS scan was followed by three MS/MS scans where the three most abundant molecular ions were dynamically selected and fragmented by collision-induced dissociation. A 55%-coverage was obtained: green shading in the deduced protein sequence indicates parts that were confirmed by peptide mapping. The arrow points at the first methionine residue in the plant protein.

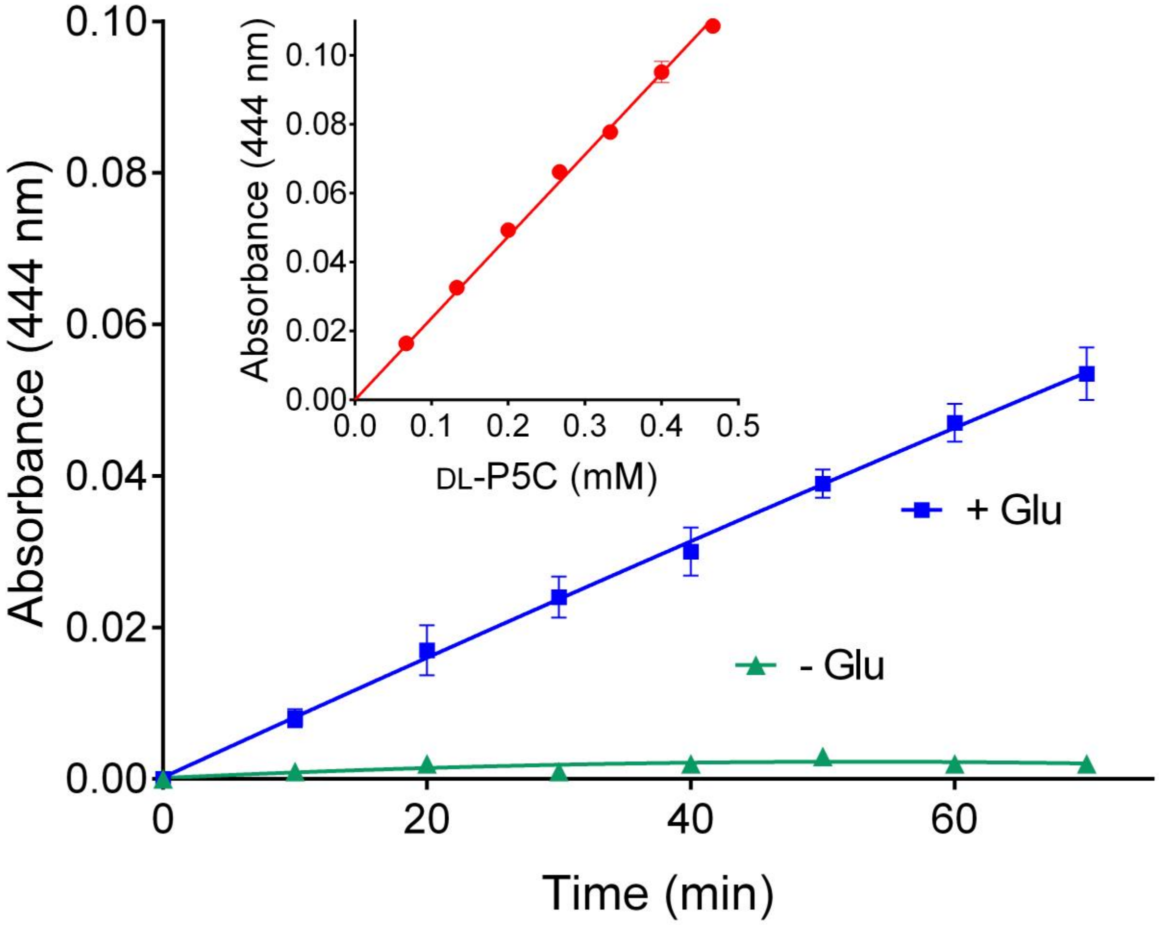

FIGURE S2 | Glutamate-dependent production of P5C by *Oryza sativa* P5C synthetase 2. The purified enzyme (2 μg) was incubated with all three substrates or in the absence of glutamate (NADPH oxidation assay). The release of P5C was verified by reaction with *o*-aminobenzaldehyde, yielding a product with an absorbance maximum at 444 nm (Williams and Frank, 1975). The inset shows a calibration curve of the colorimetric method using synthetic DL-P5C. Results are mean ± SE over 3 replicates.

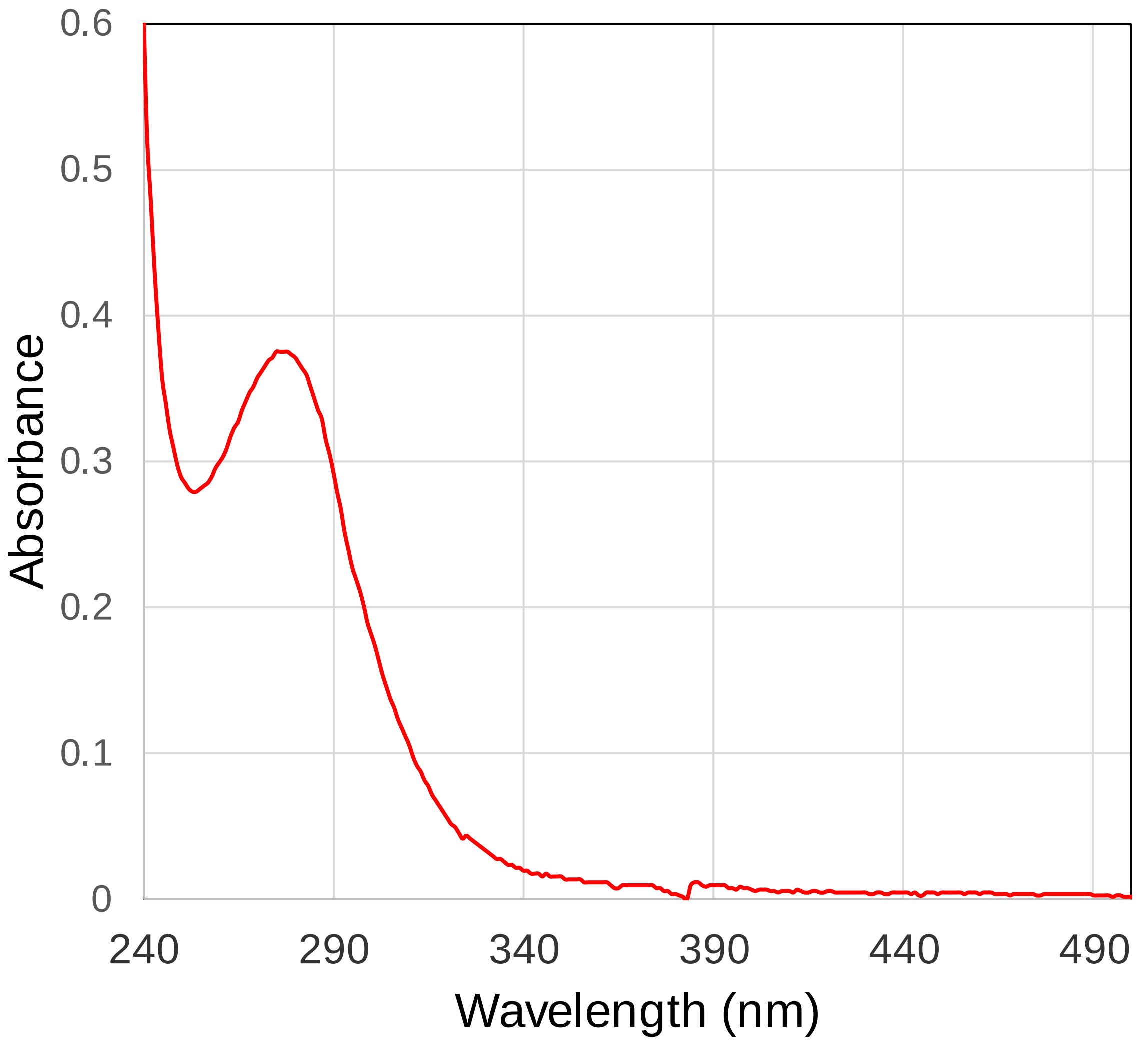

FIGURE S3 | UV-vis spectrum of *Oryza sativa* P5C synthetase 2. The concentration of the purified protein was adjusted to 40 µg mL^{-1}, corresponding to about 0.5 µM monomer.

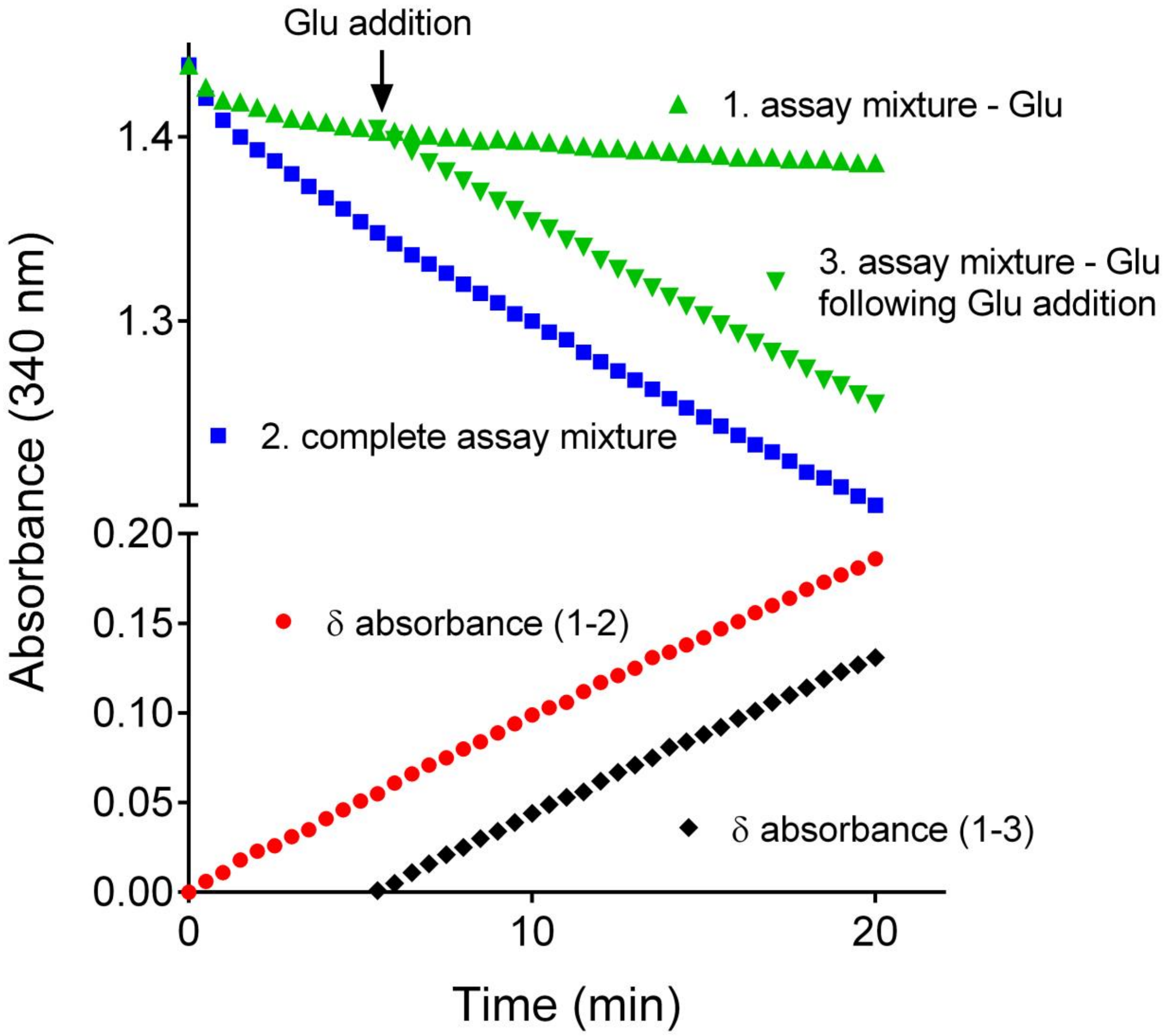

FIGURE S4 | Initial burst and stationary phase of NADPH oxidation. The purified *Oryza sativa* P5C synthetase 2 (0.8 µg) was incubated in the presence of all three substrates (2), or in a reaction mixture lacking glutamate (1). An initial burst of NADPH oxidation was evident in both cases, but in the absence of glutamate it rapidly reached a plateau. If the rate in the incomplete mixture was subtracted from that obtained in the presence of all substrate (1-2), a perfectly linear pattern was evident. When glutamate was added following a pre-incubation with NADPH and ATP (3), the rate that resulted by subtracting the resulting absorbance from that in the incomplete mixture (1-3) was perfectly comparable. Data are mean over 4 replicates, with SE never exceeding 5%.

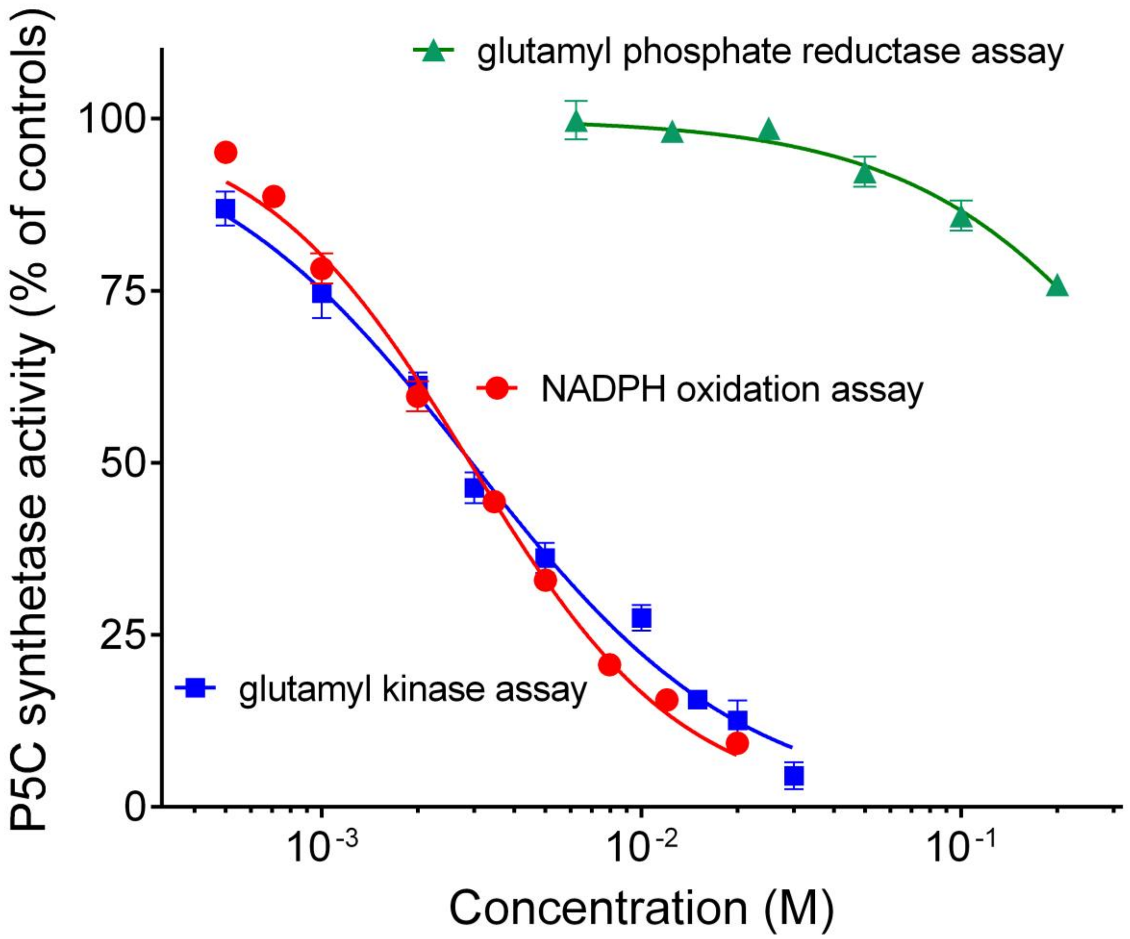

FIGURE S5 | Inhibitory effects of proline on the activity of *Oryza sativa* P5C synthetase 2. The purified enzyme was assayed with the three methods that evaluate the whole physiological reaction (NADPH oxidation assay), the phosphorylation of glutamate (glutamyl kinase assay), and the P5C-dependent, reverse reduction of $NADP^+$ (glutamyl phosphate reductase assay), respectively. Results were expressed as per cent of mean values in untreated controls. Presented data are means ± SE over three technical replicates. While the first two methods yielded similar results, the latter reaction was almost insensitive to feed-back inhibition by proline.
